# Deep Learning-Inspired IoT-IDS Mechanism for Edge Computing Environments

**DOI:** 10.3390/s23249869

**Published:** 2023-12-16

**Authors:** Abdulaziz Aldaej, Tariq Ahamed Ahanger, Imdad Ullah

**Affiliations:** 1 College of Computer Engineering and Sciences, Prince Sattam Bin Abdulaziz University, Al-Kharj 11942, Saudi Arabia; 2 Department of Management Information Systems, College of Business Administration (CoBA), Prince Sattam Bin Abdulaziz University, Al-Kharj 16278, Saudi Arabia; t.ahanger@psau.edu.sa; 3 School of Computer Science, Faculty of Engineering, The University of Sydney, Sydney, NSW 2006, Australia; imdad.ullah@sydney.edu.au

**Keywords:** AI, IDS, IoT, security, edge computing

## Abstract

The Internet of Things (IoT) technology has seen substantial research in Deep Learning (DL) techniques to detect cyberattacks. Critical Infrastructures (CIs) must be able to quickly detect cyberattacks close to edge devices in order to prevent service interruptions. DL approaches outperform shallow machine learning techniques in attack detection, giving them a viable alternative for use in intrusion detection. However, because of the massive amount of IoT data and the computational requirements for DL models, transmission overheads prevent the successful implementation of DL models closer to the devices. As they were not trained on pertinent IoT, current Intrusion Detection Systems (IDS) either use conventional techniques or are not intended for scattered edge–cloud deployment. A new edge–cloud-based IoT IDS is suggested to address these issues. It uses distributed processing to separate the dataset into subsets appropriate to different attack classes and performs attribute selection on time-series IoT data. Next, DL is used to train an attack detection Recurrent Neural Network, which consists of a Recurrent Neural Network (RNN) and Bidirectional Long Short-Term Memory (LSTM). The high-dimensional BoT-IoT dataset, which replicates massive amounts of genuine IoT attack traffic, is used to test the proposed model. Despite an 85 percent reduction in dataset size made achievable by attribute selection approaches, the attack detection capability was kept intact. The models built utilizing the smaller dataset demonstrated a higher recall rate (98.25%), F1-measure (99.12%), accuracy (99.56%), and precision (99.45%) with no loss in class discrimination performance compared to models trained on the entire attribute set. With the smaller attribute space, neither the RNN nor the Bi-LSTM models experienced underfitting or overfitting. The proposed DL-based IoT intrusion detection solution has the capability to scale efficiently in the face of large volumes of IoT data, thus making it an ideal candidate for edge–cloud deployment.

## 1. Introduction

The industrial sector has been revolutionized due to the widespread adoption of the Internet of Things (IoT). But, as a result of this expansion, attackers have become more vigilant in the pursuit of vulnerable entry points in IoT networks. Cyberattacks aimed at susceptible smart devices have increased dramatically in recent months [1]. When linked to Critical Infrastructure (CI), IoT-enabled networks are particularly vulnerable to a variety of attacks. The quality of service and security of CI systems may be negatively impacted by delays in supporting infrastructure, such as smart grids, and manufacturing. Enhanced Intrusion Detection Systems (IDSs) depend on attack signatures but also employ network traffic characteristics to identify aberrant network connections, which are necessary to determine attacks over IoT devices. Many Deep Learning (DL)-inspired algorithms have been presented to provide a solution to the IDS security issue [2]. They outperform more conventional Machine Learning (ML) methods, like Support Vector Machines (SVMs) in terms of detection performance, including accuracy (96.65%), specificity (95.45%) and recall (96.68%). Because of their computational complexity and high performance, DL methods are implemented in shared or cloud-based infrastructures [3]. As IoT data are large, they must be aggregated to centralized nodes for training DL techniques [4]; this also causes delays in the detection process. Delay-sensitive CIs make DL techniques impractical for use in intrusion detection. Distributed edge–cloud frameworks are proposed as a countermeasure [5]. They can efficiently determine attacks over the edge platform, avoiding delay in the identification of malicious actions. It is possible to scale IoT devices in large domains with faster reaction times during attacks by using edge nodes to offload compute workloads from a centralized cloud node. For the edge layer to implement existing DL-based IDS approaches, either a high number of edge nodes is needed or edge traffic is moved to central nodes to train the DL algorithm [6], both of which might raise the communication overhead. The generated DL models have greater compute and memory requirements, making it impractical to deploy them on edge nodes for efficient intrusion detection because of the associated communication overhead. To deal with the high dimensionality of the IoT dataset’s attributes, several solutions were suggested, using advanced DL techniques [7].

**Figure 1:**
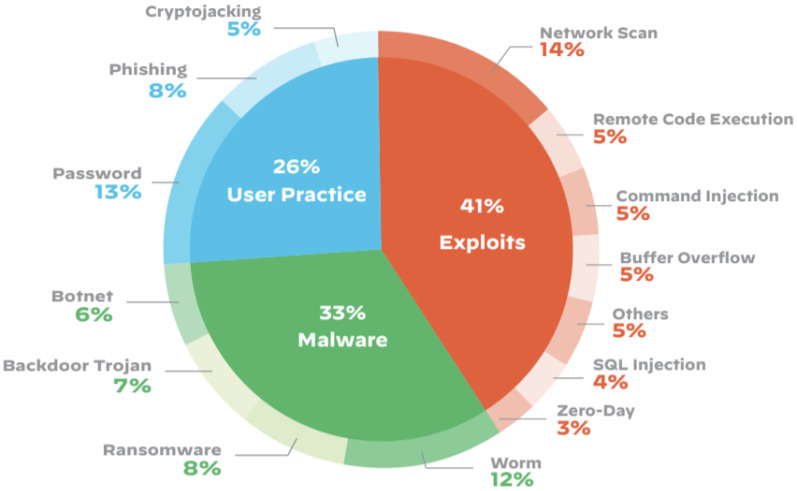
IoT attack categories.

Backpropagation-based sophisticated attribute selection algorithms, although effective, may lengthen the training process and cause delays in deployment. Henceforth, a two-stage procedure is presented to streamline the construction of the DL-inspired security framework for edge computing, which will increase efficacy for the real-time platform. Conspicuously, a multi-class issue is transformed into a binary-class problem by classifying attacks on the IoT network as time series. Figure 1 shows the IoT attack categories (Source: https://threatpost.com/half-iot-devices-vulnerable-severe-attacks/153609/ (accessed on 11 October 2023)). Then, basic attribute reduction is utilized to reduce the data needed for training the DL algorithms, including Mutual Information (MI), Group Method of Data Handling (GMDH), and Chi-Square (Chi-Sqr). An optimized technique is used for IoT attack detection, after which the reduced datasets are uploaded to a cloud node for training the DL algorithm. These procedures provide the partitioning of DL tasks and cut down on both network lag and processing time. The BoT-IoT dataset [8] is used to test the proposed technique since it includes both benign and malicious IoT traffic. The state-of-the-art primary contributions are mentioned ahead.

### State-of-the-Art Contributions

1. Detect attacks on latency-critical networks via an edge–cloud-distributed Intrusion Detection System.
2. Bifurcating temporal data into smaller subsets according to type of attack allows for the distributed analysis of a large-scale BoT-IoT dataset.
3. With the help of attribute selection methods, the proposed technique can drastically decrease the size of the dataset without sacrificing accuracy in classifying observations.
4. Recurrent Neural Networks (Simple-RNN and Bidirectional LSTM) are used in the BoT-IoT dataset to identify attack traffic and determine the performance enhancement as compared to the state-of-the-art techniques.

The rest of the paper is organized in different sections. The relevant literature on IoT threat detection is provided in Section 2. Section 3 details the proposed IDS framework, including the attribute selection methods and DL models used. Section 4 presents an experimental simulation for validation purposes. Section 5 concludes the paper with future research directions.

## 2. Literature Review

Spadaccino et al. [9] compiles a comprehensive overview of the usage of IDSs in IoT networks, including how edge computing is used to aid in IDS deployment. The authors identified novel problems that occur during IDS deployment in an edge situation and provided potential solutions. Particular attention was paid to anomaly-based IDSs, with a presentation of the primary anomaly-detection methods and a discussion of machine learning techniques and their application in the context of an IDS, including a description of the potential benefits and drawbacks of each. However, limitations include the limited evaluation on diverse edge computing environments, and lack of comparison with traditional intrusion detection methods. To protect the IoT from threats, Khater et al. [10] proposed a lightweight Host-Based IDS that employs the Modified Vector Space Representation (MVSR) N-gram and Multilayer Perceptron (MLP) model. This system was implemented with the help of Fog Computing devices. The limiting aspects included the dataset used for evaluation lacking diversity and possibly not representing real-world scenarios. The proposed mechanism’s performance on resource-constrained fog devices was not thoroughly examined. To identify malicious activity in IoT networks, many methods have been suggested. Syed et al. [3] trained and classified attacks on a BoT-IoT dataset using a Feed-Forward Neural Network (FNN). The FNN model was able to identify many types of attacks with an accuracy of 97% and a high F1 score, which is used in classification tasks to evaluate the performance of a model and its harmonic mean of precision and recall. Several types of IoT attacks, however, have poorer accuracy and recall values when using the trained FNN model. An ensemble hybrid IDS was developed by Jasim et al. [11], which combines an attribute selection step based on information gain with an ensemble of ML algorithms. The experimental findings showed that the classification techniques are much better as part of an ensemble of classifiers. As a comparison, the proposed classifiers managed 91% and 89% accuracy, respectively, whereas the ensemble classifiers obtained 98.9% accuracy. Hybrid DL was introduced by Popoola et al. [7], for which the authors suggested using a Long Short-Term Memory Autoencoder (LSTMA) layer for dimensionality reduction and then cascading that layer with a Bi-LSTM layer. The suggested method used less memory and performed better than competing methods of attribute reduction. On the contrary, attribute selection techniques based on DL may be computationally intensive. To train and identify sequential network data in the cloud, Aljuhani et al. [12] developed a bi-directional LSTM DL system. When tested on IoT datasets, the suggested method achieved a high detection rate (between 91.2% and 97.9%) in identifying DoS and DDoS attack traffic. Reconnaissance attacks and data theft were among the non-DoS traffic types for which the model fared badly.To enhance the multi-layered approach that aids in identifying intrusions for IoT network, a DL-based forensic model was suggested by Abd et al. [13]. Local and global representations of industrial IoT network traffic were captured by the proposed model’s gated RNN unit. By using a gated RNN unit in the proposed model, it is likely that the model can effectively capture both local patterns within shorter sequences of network traffic and global patterns that span across longer sequences. This allows for a comprehensive understanding of the industrial IoT network traffic and enables the model to make informed predictions or decisions based on both local and global representations. Compared to centralized DL-IDS approaches, the experiments on edge nodes showed a considerable performance boost. Nevertheless, the suggested distributed approach needs a large number of edge nodes to significantly enhance detection accuracy, and its performance may degrade under heavy loads. Intrusion detection in edge-based IoT systems was suggested by Guo et al. [14].

In the first phase of the two-stage detection procedure, network traffic is binary classified using K-Nearest Neighbor (KNN) and a Deep Neural Network (DNN). Specifically, the input is classified as non-invasive or intrusive by the DNN model. Instances that DNN fails to correctly classify are used by KNN, which uses the Euclidean distant to determine similarity and provide a suitable class. The key aspect is that it has to be verified for its efficacy on IoT datasets before it can be fully adopted. The suggested strategy has been tested on non-IoT datasets. Moreover, the success of the kNN method is very sensitive to the selection of the k value. Song et al. [5] suggested an ensemble learning-based method for distributed anomaly identification in edge networks. The suggested method employs an IoT–edge–cloud architecture and Gaussian mixture-based correntropy to identify attacks on the IoT. The weaknesses include the need for attribute engineering, the use of shallow ML techniques, and the evaluation of the proposed model on datasets that are not related to the IoT. To identify cyberattacks in the Internet of Medical Things, Nayak et al. [15] suggested an edge–cloud architecture using an ensemble learning technique. In addition to requiring a time-consuming attribute engineering phase for training the algorithms, the method relies on three shallow ML algorithms for ensemble learning. Table 1 provides a summary of the literature, proposing IDS approaches for IoT networks.

### Research Challenges

There are several obstacles to overcome when using DL models for IoT intrusion detection. The key difficulties are the computed complexity, time delay, and bandwidth needs, as well as properly dispersing the detection duty to several worker nodes. Therefore, the partitioning of multi-category IoT datasets is performed in distinct class data according to the time of arrival. To further minimize the size of the dataset, attribute selection is applied to each binary class dataset separately. Finally, in the current paper, DL models are utilized that can sort the data into regular or offensive kinds.

## 3. Proposed Model

**Figure 2:**
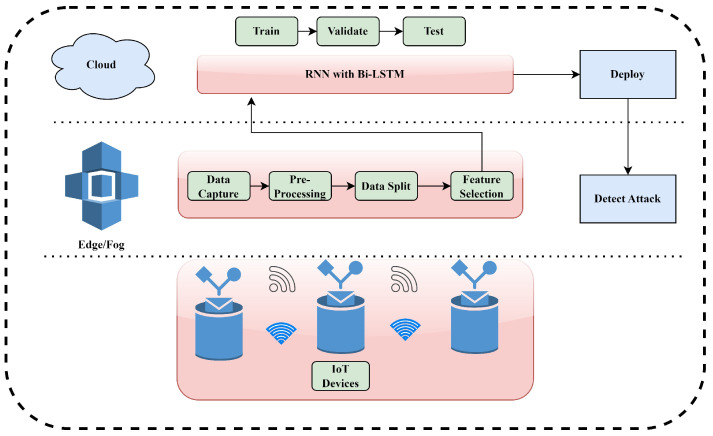
Framework workflow.

Figure 2 shows the proposed edge–cloud-based infrastructure for monitoring IoT traffic for anomalies. Gaining access to data, pre-processing, attribute identification, training, validation, and testing with the help of IoT intrusion detection models are deployed after training using DL techniques. The proposed framework has three components, including IoT devices, an edge layer, and a cloud layer. IoT devices function and communicate on the most fundamental level. Deployed at the edge layer, tools like TCP collect raw network traffic from these IoT devices. In addition, the packets are transformed into time series data at a pre-processing stage. The BoT-IoT dataset, having a wide variety of attack timestamps, is utilized. Analysis of the timestamps reveals that the vast majority of attacks have been recorded at discrete times, whereas normal data appear across the board. Henceforth, it is recommended to partition the dataset into attack-specific subsets. Therefore, training, testing, and validation on time-stamp-based dataset partitioning are performed. In addition to facilitating the distributed processing of datasets, the proposed approach employs an attribute selection methodology to drastically reduce dataset sizes before sending them to cloud instances for training. Then, the dataset is shrunk by attribute selection at the edge layer and then transferred to the cloud. DL models may be taught and tested with the help of the plentiful computational resources offered by the cloud layer. At last, the effective DL models for intrusion detection are deployed in the edge layer. Moreover, the Group Method of Data Handling (GMDH) [16], Mutual Information (MI) [17], and the Chi-Sqr statistic techniques [18] are used to evaluate the effectiveness of dimensionality reduction algorithms. Furthermore, RNN and a subclass of RNN called Bi-directional LSTM are incorporated to evaluate the attack classification performance. The detailed procedure is discussed ahead.

### 3.1. Attribute Selection

Identifying the vital characteristics and discarding non-vital ones is a crucial part of developing a reliable framework for network intrusion detection. Using the attribute selection step before training a DL model can reduce the dataset size and the amount of computing power needed to train the model. The following sections provide further detail on the different attribute selection methods utilized in the current study.

#### 3.1.1. Group Method of Data Handling (GMDH)

The GMDH [19] is an early example of a feedforward network for DL. It is part of a heuristic family of algorithms that, by identifying correlations between input attributes, may automatically create self-organization models of optimal complexity. The algorithm then forms its internal structure without any input. The Ivakhnenko polynomial [20] is used to describe the connection between the input variables y_1_ and y_2_:

(1) $$z=b+\sum_{j=1}^{n}c_{j}y_{j}+\sum_{j=1}^{n}\sum_{k=1}^{n}d_{j}ky_{j}y_{k}+....$$

Each neuron layer has *n* variables, and *b*, *c*, and *d* are respective weights in the aforementioned equation. The GMDH algorithm uses inductive learning to infer the connections between variables with optimal complexity by mimicking the natural evolution process. Input connections are simplified so that the algorithm may derive more complicated relationships. Instead of the standard m input variables, the algorithm is given n(n − 1)/2 to predict z. In addition, the computational burden is decreased by eliminating variables or attributes that are correlated with the output. For choosing the best attributes, GMDH considers all possible pairings of input attributes, with “best” referring to the most significant correlations between the input and output vectors. The following procedures are required to implement the GMDH algorithm:

1. Step 1: Two attributes are chosen at random and supplied into a single neuron.
  Step 1.1: Define the pool of attributes.
  Step 1.2: Determine subset size = 2.
  Step 1.3: Random subset selection.
  Step 1.4: Evaluate subset performance.
  Step 1.5: Select optimal subset.
  Step 1.6: Repeat the process.
2. Step 2: The weights are estimated by comparing the training set to the current state of each neuron.
3. Step 3: Probabilities are computed using the training and validation datasets at each neuron.
4. Step 4: The most effective neurons are chosen according to some objective standard.
5. Step 5: Validation error, bias error, validation, and bias error are the available criteria offered by the Python version of GMDH. In the current scenario, a validation error is selected.
6. Step 6: Users have the option of customizing neurons for every layer or having it determined automatically based on input variables.
7. Step 7: In the event of a validation error, reaching the maximum number of layers, or selecting a single neuron, the process will restart from the beginning.

In the current study, the GMDH technique is used to identify attributes, then feed those attributes into a DL model as inputs. The GMDH method is measured against both linear and covariance functions. As indicated in the equation ahead, the linear function accumulates variables linearly with the corresponding weights:

(2) $$z=x_{0}+x_{1}y_{1}+x_{2}y_{2}$$

where linear convergence includes y_1_ and y_2_, the covariation of input variables and weights (x) as depicted ahead:

(3) $$z=x_{0}+x_{1}y_{1}+x_{2}y_{2}+x_{3}y_{1}y_{2}$$

#### 3.1.2. Mutual Information

One popular metric for selecting attributes based on their quality is Mutual Information (MI). To find the most useful subset of characteristics, attribute selection algorithms use this strategy. How much information an attribute gives about the outcome and how independent it is from other characteristics are two factors in the goodness meter. MI is based on Information Theory concepts and measures the degree of dependency between two random variables. MI gives a measure of information about Z rather than only detecting the linear connection between Y and Z as would be the case with a simple linear regression analysis. Hence, MI of Y and Z is defined as

(4) $$J\left(Y:Z\right)=\sum_{z\to Z}^{}\sum_{y\to Y}^{}q\left(y,z\right)*log\frac{q(y,z)}{q(y)q(z)}$$

where the joint distribution of Y and Z is denoted by q(y, z). Both q(y) and q(z) represent the marginal probability distributions of Y and Z, respectively. The definition in terms of entropy G(.) is as follows:

(5) J(Y:Z)=G(Y)−G(Y/Z)=G(Z)−G(Z/Y)=G(Y)+G(Z)−G(Y:X)

where G(Y/Z) and G(Z/Y) are conditional entropies, and G(Y:Z) are the joint entropy of Y and Z. The MI measure of both variables is independent.

#### 3.1.3. Chi-Square (Chi-Sqr)

The independence of two occurrences or two traits may be determined with the use of statistical tests. This method of attribute selection calculates the observed and expected values of an attribute to determine whether it is effective in differentiating the target class characteristic as illustrated ahead:

(6) $$\chi^{2}=\sum_{j=1}^{o}\frac{{\left(P_{j}-F_{j}\right)}^{2}}{F_{j}}$$

where P_j_ is the actual frequency of a property and F_j_ is the predicted frequency. The Chi-Sqr statistic quantifies the absence of independence between attribute g and output class d, where g and d are instances and attributes of a dataset, respectively. Variables can be represented as the field length or size of attributes to distinguish between benign and malicious traffic aimed at IoT devices. At times of attack, frame lengths are distributed differently than they are during regular traffic. This may help distinguish between malicious and benign IoT communications. The MI with output (Z) may be calculated using heterogeneous length measures in the baseline and attack phases. Both MI and Chi-Sqr are examples of attribute selection algorithms. Attacks against IoT devices and applications may be detected by paying attention to other characteristics, including size value and header length. The reason behind this is that when creating an IoT application, the device is pre-programmed to deliver messages of a certain size. Hence, irregular connections in IoT settings may be detected by measuring packet and field durations that deviate from the norm. Chi-Sqr attribute selection uses a hypothesis assessment to choose the most relevant characteristics with independence scores over a threshold. Hence, characteristics that do not rely on the target class contribute little to the classification of the instance and have low Chi-Sqr scores as a result. In a similar vein, MI chooses characteristics that tell us the most about y, the outcome variable. The Scikit learn [21] SelectKBest library was used to find the top K attributes that have a significant correlation with the final metric (MI and Chi-Sqr, respectively). To provide a level playing field with the GMDH technique, which chooses fewer than 15 attributes for all classes of the dataset, the value of K is set at 15.

#### 3.1.4. Selection Validation

To find the best characteristics for DL, researchers used a four-stage experimental design. First, several types of sub-datasets are formed, and then 20% of the dataset is taken out for ranking and selecting attributes. As computing time is expensive, this would help speed up the process of determining which attributes are the most useful. Third, the sampled dataset is used as input for the proposed algorithms. The best characteristics of the dataset were analyzed using the GmdhPy package, which was used here with its default settings. For both development and testing, we used the 65–35% split, which is GmdhPy’s default. Negative values were eliminated by performing min-max scaling on the input data for MI and Chi-Sqr. The K-Nearest Neighbor technique is used to calculate entropy as part of the MI algorithm in sci-kit learn, a nonparametric approach discussed in [22]. To learn the boundaries between classes, a DL algorithm is given the trimmed-down dataset after attribute selection is performed. The following sections provide further detail about comparing DL algorithms.

### 3.2. RNN

**Figure 3:**
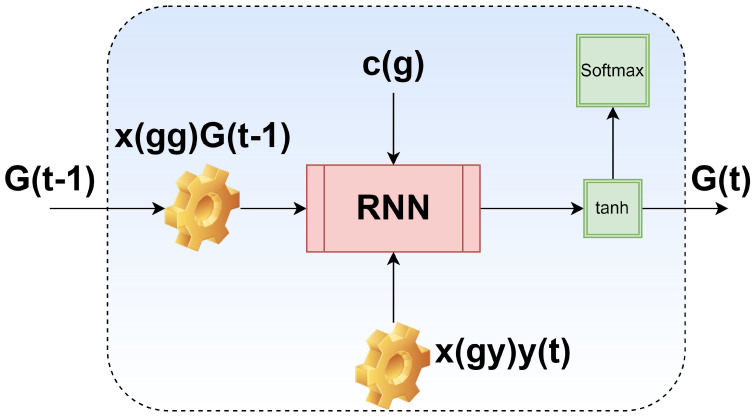
Single RNN cell.

**Figure 4:**
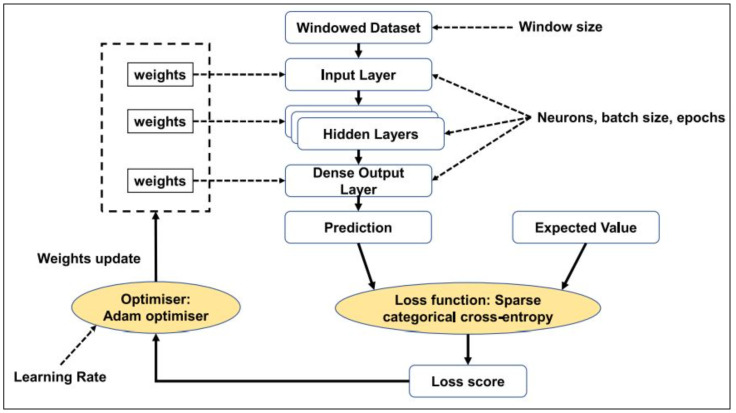
Proposed RNN framework.

**Figure 5:**
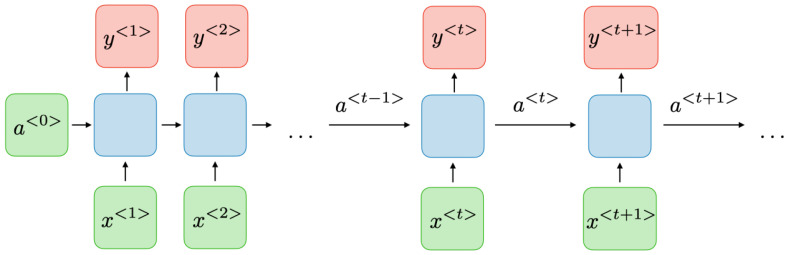
Generic RNN framework; variables are defined accordingly.

By implementing information flow in loops from the present state to the past states, RNN enables the persistence of information. Because of their ability to take into account the historical status of the network traffic to predict the present state, RNNs are well suited to the task of detecting network intrusions [23]. Each cell in an RNN has one input with one internal stage, and they propagate from one cell to the next at each time step. Certain data are sent from one time step to the next through the activation of the hidden layer. To fully process the input data, an RNN loops over Tn time steps, each of which involves computing the attributes of the previous time step. X^t^ is the current data input, y denotes the input value of parameter at time t, and g${}^{t-1}$ is the prior hidden state that stores historical data in a single RNN cell design. In Figure 3, the basic structure of a single RNN cell is presented. Figure 4 provides the generic RNN model and Figure 5 represents the Generic RNN framework and associated variables are accordingly. The hidden state g${}^{\prime }$ is calculated as:

(7) $$g^{\prime }=H\left(X_{gg}g^{t-1}+X_{gy}y^{t}+c_{g}\right)$$

where H is the activation function in the hidden layers, X is the associated weights, and c is the bias. Moreover, the prediction can be performed as z^t^ = softmax(X_zg_ + c_z_), a combination of RNN cells based on temporal steps.

### 3.3. Bi-LSTM

**Figure 6:**
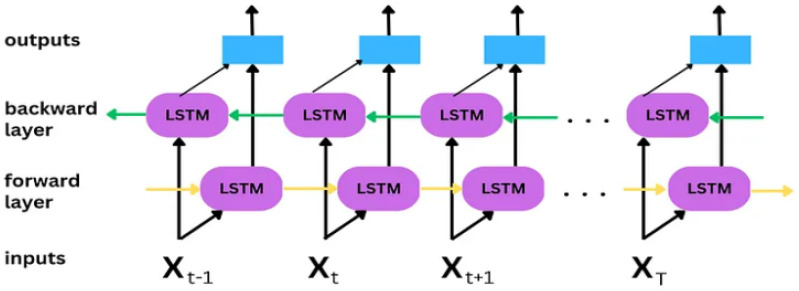
Generic Bi-LSTM framework; variables are defined accordingly.

LSTM represents an enhanced DL technique that takes into account long-term dependencies to forecast the output class. Hence, LSTM has a greater capacity for long-term memory, allowing individuals to make more informed choices. RNNs have trouble learning long-term dependencies because of the time lag between receiving input and making a decision, a phenomenon known as the vanishing gradient issue. To resolve it, LSTM uses gates to forward data to the appropriate cells and to store context data for longer. Adding input from both the forward and backward directions into an LSTM hidden layer is a great way to boost its performance (Figure 6).

### 3.4. Completion Time

Various phases of detection were analyzed to obtain delay for the suggested IDS method. Pre-processing, decomposing time series data, selecting attributes, and running the DL model are the primary procedures that need computational resources. The packet capture tool PCAP is assumed to include O attributes and O samples. The complexity of the pre-processing step is O(O) since it must take into account all O samples for all O characteristics. During the attribute selection phase, three competing techniques are evaluated concerning their respective time complexity. The complexity of the ultimate detection method will vary depending on the chosen algorithm. The temporal complexity of the GMDH algorithm is derived. Time complexity should primarily focus on stages 2, 3, and 6 of the GMDH. Probabilities are determined by combining the training and validation datasets, and an initial set of L models is constructed using a training set of T samples (T < N). It has a temporal complexity of O(L^2^). The proposed procedure involves applying an external selection criterion to the original models and ranking them accordingly. When R_l_ are the created candidate models from the input characteristics, the temporal complexity of this operation is O(P_l_ ∗ logP_L_). Then, until a halting criterion is met, steps two and three are repeated. If the whole GMDH network has m layers, the best neurons/models from each layer are promoted to the next. The maximum computational time of GMDH-specific attribute selection is O(M ∗ P_l_∗ logP_L_), where N is the maximal layers and l is the initiating modules. The computational time for MI-specific attribute identification is O(NO) for N samples and O attributes due to the necessity to compute joint entropy of attribute-to-category mapping. The Chi-Sqr statistic has a time complexity of O(S ∗ N^2^). S represents the total number of random permutations, and N represents the total number of samples. The suggested method concludes with a study of the classification border between target classes using DL models. O(X), where X is edge nodes, is the temporal complexity for LSTM networks. RNN and LSTM networks have O inputs, Z outputs, and G hidden layers, where each edge represents a neuron in the network. As the RNN weight calculation difficulty is given by

(8) $$X=OG+G^{2}+GZ$$

the LSTM weight calculation complexity is given by

(9) $$X=4OG+4G^{2}+3G+GZ$$

We simplify the DL process by using attribute identification and converting a multi-category output to the specific category. To further improve the performance of the DL models, hyper-attribute tuning is used to determine the optimal neuron count.

## 4. Experimental Implementation

This section provides the experimental simulation of the proposed technique for validation purposes. Moreover, the performance enhancement is estimated based on the comparative analysis with state-of-the-art research works.

### 4.1. Conception of Experiments

Experiments were performed on a high-performance computing cluster equipped with 16 GTX 2160 Ti GPU powered by Intel Gold at 3.10 GHz and 512 GB of memory. The RNN and bidirectional LSTM components were implemented using the TensorFlow library and the Keras libraries. A total of 65% was set aside for training, 15% was used for validating the models, and the remaining was used in testing. The 70/30 rule was used to divide the dataset into training and testing sets, which is consistent with the Pareto principle. The remaining 65% and 15% of the training set were used for validation. The validation set helps evaluate trained models objectively and gives guidance throughout the training process.

### 4.2. Dataset

To estimate the effectiveness of the intrusion detection mechanism, the BoT-IoT dataset was used. The dataset was chosen because it includes both malicious and benign IoT traffic in large quantities. DDoS, DoS attacks across UDP, TCP, HTTP, data exfiltration, reconnaissance traffic, and the keylogging attack are the attack types. In Table 2, different types of attacks are shown in the BoT-IoT dataset, along with the times when they occurred. By splitting the cases into sub-classes comprising just one attack type and regular traffic, the dataset is transformed into a binary classification. The process of translating raw data into attributes at the datum level is performed. IP, Frame, UDP, and HTTP fields were employed among the total of 30 used as attributes. To train the RNN DL system, we next had to transform the data collection into a temporal segments. The arrival time of the frame was determined by querying the epoch property. After obtaining the timestamps, the data underwent pre-processing, which included operations such as embedding to encode the categorical data. In particular, categories existing in the dataset were used to encode port variables. Sub-datasets, each including just attacks of a certain kind and normal occurrences as background traffic over the same period, were then created by separating and sorting individual attack instances based on packet timestamps. First, the BoT-IoT dataset is transformed into a csv file with packet-level information. By taking into account the packet arrival timings, the dataset is then transformed into a time series. Sub-datasets are created for each attack type and organized by period, with typical occurrences in the same period included. The next phase is pre-processing, which involves the elimination of duplicates and the encoding of categorical data, such as the HTTP method. Finally, occurrences are normalized. Table 3 displays the number of cases and characteristics retrieved for each class across all periods. Dual category data are gathered and incorporated for attribute identification, and subsequent training, validating, and testing of the DL model is performed, one for each attack category, and cases are seen throughout its time.

### 4.3. Model Formulation

We utilized the best-ranked attributes to construct a model in Keras’ RNN implementation. As the input shape is affected by the attack category, the attribute for the proposed framework is determined accordingly. For the service attack category, 92 attributes, with a 3 s window, and 512 neurons result in 23,698 training attributes. Using the GMDH attribute selection approach, training attributes are reduced in the RNN initial layer. The Bi-LSTM framework was developed with the use of the Keras DL package. The activation functions tanh and softmax were selected for the hidden and dense layers of RNN and BiLSTM, respectively. Using the Adam Optimizer, we decided to use accuracy as our measure of choice and sparse categorical cross entropy as our loss value. The proposed framework is depicted in Figure 4. First, the BoT-IoT temporal data segments were transformed in segments, which use a predetermined amount of temporal instances to successively cover the complete time series. The size option determines how many time samples are used to create a window. The input characteristics are collected by an input layer in the architecture. Next, the calculation is carried out by a hidden layer made up of many neurons, and the output layer is responsible for sorting occurrences into normal and attack categories. Interconnecting weights were computed and fine-tuned across the three layers of the DL network during training. The backpropagation technique is used to update and choose the interconnection weights that result in the lowest possible loss.

### 4.4. Adjusting Hyper-Attributes

It was determined by experimenting with different values for RNN hyper-attributes which settings were most suited for the model suitable. Hidden layers, learning rate, dropout rate, batch size, neuron count, epochs, and window size were all taken into account during tuning. The starting point was an RNN model with three dense layers and a 512 neuron count in the first two dense layers, trained using the Adam optimizer’s default learning rate of 0.002. Table 4 displays the range of hyper-attributes that was tested. The results show that adding more hidden layers to RNN improves the model’s performance but that adding more than three hidden layers has no noticeable effect on performance, hence three hidden layers were used here. The optimal performance across all classes was achieved by using 512 neurons. No more tweaking was performed since the dropout rate did not influence the model’s performance. The Adam optimizer’s learning rate had a significant effect on performance, with a value of 0.0002 producing the best results. This was the value used for model training. Most iterations terminated before 19 epochs; therefore, increasing the number of epochs did not improve the model’s performance. To avoid the overfitting issue, we included an early stopping condition with four iterations, which would terminate the train procedure if validating loss remains fixed or rises with subsequent iterations. The learning process speed is affected by the batch size when working with huge datasets. The testing showed that the accuracy of RNN was enhanced by increasing the batch size to 64. To generate window data segments, the size of the window must be specified; the results indicate that the optimal window size is 4. Specifically, simulations were performed for window sizes 2, 4, 8, and 16. Based on the accuracy acquired, window size 4 was selected. Moreover, window size 2 registered an accuracy of 82.65%, window size 8 registered an accuracy of 81.01%, and window size 16registered an accuracy of 78.15%. When it came to Bi-LSTM, similar hyper-attribute measures were used since they resulted in better model performance. But instead of 25, RNN now uses batches of 60. The RNN network was designed with three hidden and a dense layer, whereas the Bi-LSTM network had two hidden and a dense layer.

### 4.5. Evaluation of Outcomes

We give the evaluation findings, which detail how well the RNN and bidirectional LSTM models performed, as well as the characteristics those algorithms chose to use. Each attribute selection algorithm’s findings are displayed, together with the amount of data reduction that happened, thanks to the best-picked attributes. It allows for a thorough assessment of the attribute selection outcomes. Confusion matrices are used to evaluate how well a model can distinguish between normal and attack classes in a given subset of the network traffic dataset. Accuracy, recall, precision, and the F1 score are four more measures used to evaluate performance. Specifically, the detection performance was measured in terms of the accuracy, precision, recall, F1 score, and area-under-curve metrics.

#### 4.5.1. Data Reduction and Attribute Selection

Table 5 shows the dramatic decrease in data size that occurred from attribute selection using the three techniques. The service scan category had the greatest decrease in data size, measured in MegaBytes (MB). In this subcategory, the reduced data storage space is used for GMDH, MI, and Chi-Sqr, whereas the entire dataset required 1022 MB of storage space. For data theft, DDoS-HTTP, keylogging, and DoS-HTTP, the GMDHlr and GMDH-lr-cov approach reduced data size by over 85%. The data size was reduced by between 85% and 95% after selecting 10 characteristics for both MI and Chi-Sqr. Our findings suggest that, by filtering out irrelevant and uncorrelated information, attribute selection approaches may significantly cut down on the quantity of data needed to train and assess DL models. Table 5 lists the top ten characteristics chosen by each separate algorithm. The abductive network training technique GMDH chooses the attribute sets’ best predictors as the model complexity rises. Using this method, high-dimensional BoT-IoT datasets may be represented in a low-dimensional attribute space that nevertheless captures their essential characteristics. As compared to the non-linear covariance function employed during GMDH classifier training, the findings of the attribute selection suggest that the latter selects fewer attributes, allowing for more data reduction. This could be because of the relatively linear connection between the dataset’s input variables and its output class variables. Low-dimensional representations of the original input attribute space are sought by attribute extraction methods, like Principal Component Analysis and embedding approaches, but these methods are difficult to understand. On the other hand, GMDH creates candidate models and picks intermediate models of increasing complexity depending on preset selection criteria, like validation or bias error, number of neurons, and the maximum number of layers. By doing so, GMDH can objectively choose attributes, picking the characteristics that have the most bearing on the goal objective automatically based on the finest examples of those attributes.

#### 4.5.2. Efficiency of the Model

**Figure 7:**
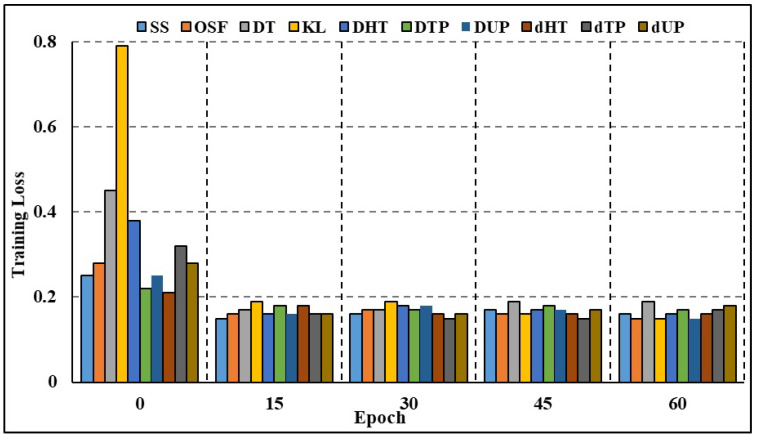
Training loss comparison: all categories.

**Figure 8:**
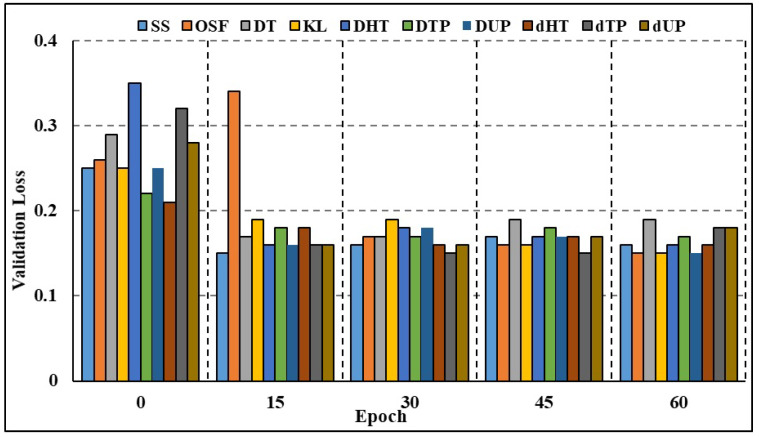
Validation loss comparison: all categories.

**Figure 9:**
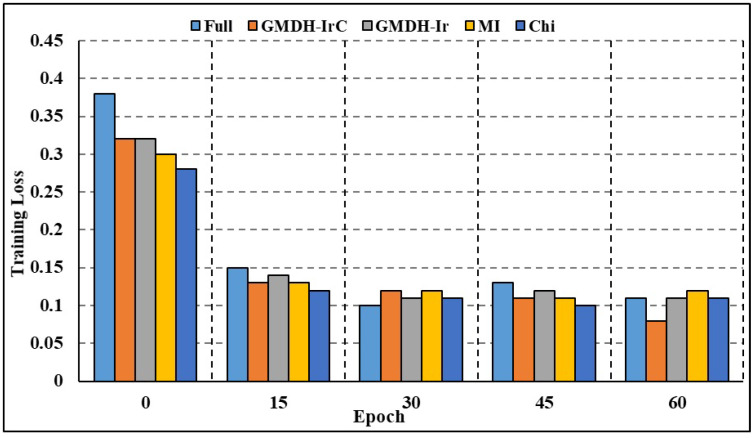
Training loss comparison: Bi-LSTM for service-scan attack.

**Figure 10:**
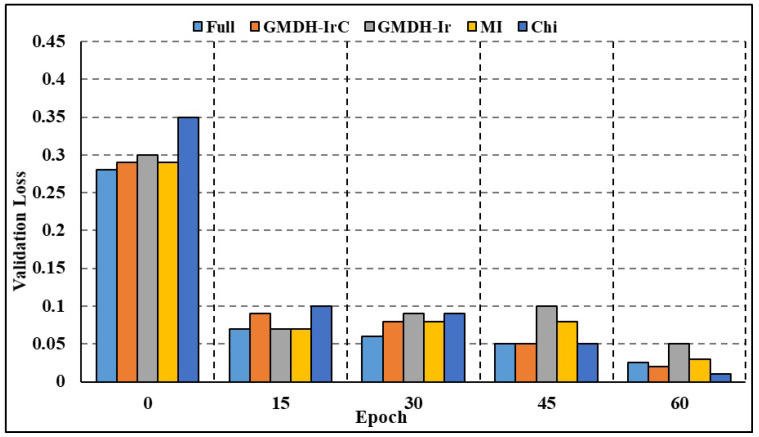
Validation loss comparison: Bi-LSTM for service-scan attack.

The effectiveness of both full-attributed and attribute-selected RNN and Bi-LSTM models was compared over the BoT-IoT dataset and NSL-KDD dataset. The NSL-KDD dataset (Source: https://www.unb.ca/cic/datasets/nsl.html (accessed on 11 October 2023)) is a more recent version of the well-known KDD Cup 1999 dataset. It is a benchmark dataset for network-based Intrusion Detection Systems, and it includes various types of attacks and normal network traffic. RNN studies on different attack traffic subsets reveal that attribute-selected models outperform and are on par with complete attribute-based models. Experimental results show that compared to models trained on the whole attribute set, those trained on the limited set of attributes resulted in greater recall rates. Nevertheless, models trained on characteristics picked by the GMDH approach have reduced accuracy compared to other models in a few categories of attacks, including OS fingerprinting, Keylogging, and DoS-HTTP. For the classification task comparison, the DL models were also compared to other machine learning models. The models were compared using a binary classification on the complete dataset without any time-based data partitioning or attribute selection. According to Table 6 (BoT-IoT) and Table 7 (NSL-KDD), RNN and Bi-LSTM models outperformed other popular DL algorithms, including Naive Bayes (NB), Random Forest (RF), and Support Vector Machines (SVMs). In addition, training using limited subsets of data points significantly increases the time required for SVM to converge and finish the training process. Table 8 shows the comparative analysis of the suggested method against various IDS frameworks. According to the findings, certain frameworks may be more accurate overall but at the expense of precision and recall. The accuracy, F1 score, and recall rates for the suggested framework presented in [24] are excellent. To achieve quicker and more accurate edge detection, however, our work focuses on reducing the number of attributes in DL models and creating class-based sub-datasets for distributed processing. Both full-attribute and attribute-selected Bi-LSTM models produced the same result. Experimental results show that compared to the model trained with all available attributes, the performance metrics for models trained with a subset of attributes are better. The Bi-LSTM loss throughout training and validation is shown in Figure 7 and Figure 8. Both figures show that for the vast majority of sub-datasets, training and validation lasted for more than 25 epochs but less than 60 epochs. At the third epoch, the training loss decreased below 0.1 across the board for attack sub-categories. Moreover, the validation loss decreased to below 0.05 across all categories, ranging from 0 to 0.02 for DoS HTTP and DoS UDP, respectively. As it can take into account long-term temporal dependencies while generating choices, the Bi-LSTM model also exhibited overall superior performance than the RNN models. Overfitting and underfitting may be problematic in intrusion detection tasks, but the models trained on decreased attribute space demonstrated resistance to both. Training and validation loss for both the whole and restricted attribute space of a single kind of attack are compared in Figure 9 and Figure 10. Models trained with a smaller attribute space did not overfit or underfit as measured by the training and validation losses, which stayed between 0 and 0.01 after the first 13 epochs.

### 4.6. Comparative Analysis

**Figure 11:**
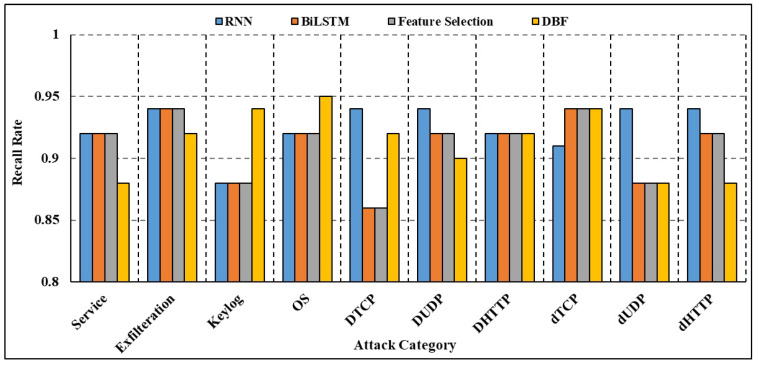
Comparison of recall rate.

**Figure 12:**
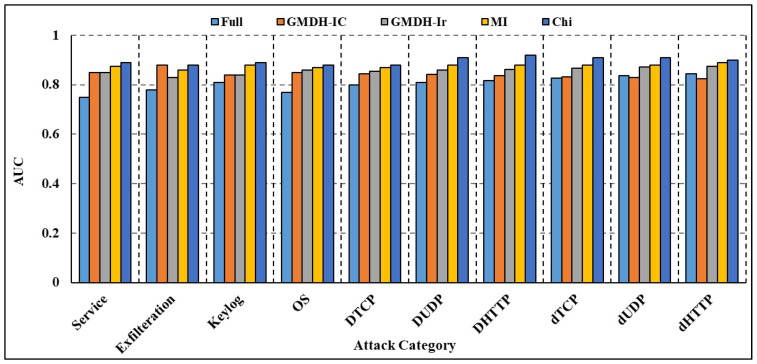
Comparison of AUC of proposed RNN.

**Figure 13:**
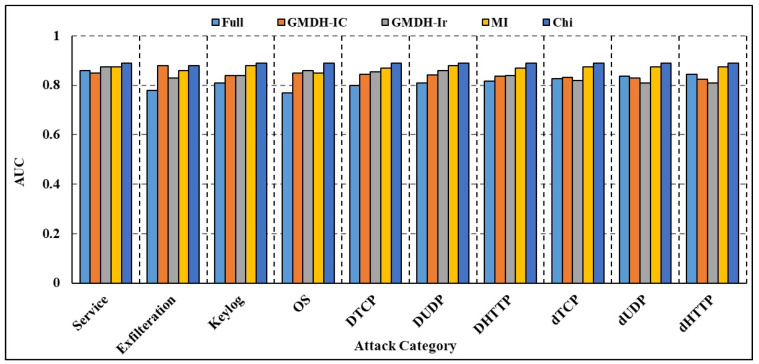
Comparison of AUC of proposed Bi-LSTM.

The findings of the deep blockchain framework (DBF) presented in [25], which uses Bi-LSTM to categorize attack traffic in the BoT-IoT dataset, were compared with those of our own suggested method. Figure 11 gives a comparison of the recall rate of several attack sub-categories. We found that our strategy for identifying attack traffic had a greater recall rate than theirs did. Service scanning, operating system fingerprinting, data exfiltration, and keylogging are just a few examples. The attribute selection procedure enhances the efficacy of DL models in identifying IoT-based threats as shown by the results of attribute selection and DL-based categorization. Using an attribute selection step with DL has the primary benefit of drastically shrinking the dataset without sacrificing any of the useful class-discriminating information between the input and output variables. As compared to other attribute selection algorithms, the MI algorithm’s choices resulted in the greatest performance gains across several types of attacks. The area-under-the-curve metrics for each of the subcategories is shown in Figure 12 and Figure 13. If a model has a higher AUC, it suggests it does a better job of predicting what category each data point belongs to. For simplicity, we only provide the total AUC score when there are numerous types of attacks. Using attribute selection helps the deployed model consume fewer computing resources. Using the stored DL models, the number of Floating Point Operations Per Second (FLOPS) needed was estimated to compare the computational needs of the models with full and reduced attribute sets. Multiplication, addition, and other batch normalization and activation function operations are all part of the FLOPS that are produced. Table 9 details the FLOPS demands of the created models for different types of attacks. Empirical findings show that, across all categories, models trained with a subset of attributes need around 0.20 million fewer FLOPS. Hardware devices used at the edge and edge layers, which generally allow 1–99 and 100–999 million FLOPS correspondingly, are likewise compatible with the FLOPS recorded for the proposed technique. Nevertheless, following attribute selection, all models trained on the smaller dataset recorded fewer than 0.62 million model attributes, using less than 2 MB of memory. Nevertheless, low-powered IoT devices may not be able to accommodate such memory utilization due to memory limits on the order of Kilo Bytes. In comparison to micro-controllers, the memory and processing power of most devices at the edge layer—such as access points, tiny servers, routers, gateways, and so on—is far greater. As a result, the proposed method is well suited for DL-based intrusion detection applications at the edge layer since its low FLOPS and memory needs allow for faster detection times. To improve generalization performance, the suggested method may also be used in settings where attack detection is dispersed across fewer processing nodes in the edge layer. As a result, fewer worker edge nodes and fewer computational resources are needed for intrusion detection. In addition, a cloud–edge intrusion detection framework is provided to correctly identify attacks on IoT devices by reducing the dataset size using attribute selection and outsourcing the time-consuming and difficult model training activities to the cloud nodes. The process of creating IDS may be considerably improved by adding an attribute selection phase before the DL layer. This is because experts have figured out which characteristics are crucial for spotting attacks.

One of the disadvantages of using a subset of the dataset is that the accuracy value for certain categories is lower than it would be using the whole dataset. To get around this restriction, an ensemble of classifiers that have been trained on carefully chosen attributes may be constructed.

## 5. Conclusions

DL-inspired IDS have been found to successfully recognize attack patterns. DL-based IDS needs to be put closer to the IoT devices to decrease the time delay in detecting sophisticated attacks targeting the IoT paradigm. The adoption of DL-based IDS near-the-edge IoT devices may be hampered, however, by issues such as the vast amount of IoT data and the sophisticated computing needs of DL approaches. Conclusively, we present a DL-based IDS framework for the IoT that may be efficiently implemented utilizing an edge–cloud design. The suggested system includes partitioning the dataset based on the time of arrival of the attack flow. The high-dimensional BoT-IoT dataset is further reduced in size by selecting the characteristics of interest. Hybrid RNN algorithms are trained and tested on the reduced dataset to categorize the occurrences as an attack or regular traffic. Our findings demonstrate the usefulness of our suggested framework and its resistance to over- and underfitting. The dataset size was decreased by 85% due to the attribute selection phase, which means that vast amounts of IoT data may be sent to the cloud network for DL tasks with far less impact on the network’s latency. Both the RNN and Bi-LSTM models, when trained on a smaller attribute set, outperformed conventional techniques. In addition, the attribute selection process minimized the time and space needed to train DL models. The proposed DL-based edge–cloud IDS method is enhanced in detecting cyberattacks targeting the IoT devices in CI due to its ability to split datasets, reduce dataset size through attribute selection, reduce the computational requirements of trained models, and provide superior detection capability.

## Figures and Tables

**Table 1:** Comparative analysis.

Ref.	Technique	Performance	Data Split	Data Reduction	Layer	Dataset
[12]	CorrAUC based attribute selection	Low precision	NA	Yes	Centralized	BoT-IoT
[14]	DL techniques using GRU	Achieves better overall performance	NA	Yes	Edge-based, Distributed	BoT-IoT and UNSW-NB15
[15]	DL-inspired feed-forward technique	High classification precision	NA	NA	Centralized	BoT-IoT
[13]	DL framework with BiLSTM model and blockchain technique	Enhanced accuracy	NA	NA	Centralized	UNSW-NB15 and BoT-IoT
[3]	LSTM	High recall rate	NA	NA	Cloud	ISCX2012
[11]	Uses multi-modal with auto-encoder	Improved F1 measure	NA	NA	Distributed	BoT-IoT
[7]	RNN-based attack identification	Non-IoT-based traffic	Yes	NA	Centralized	NSL-KDD

**Table 2:** BoT-IoT dataset: time analysis.

Category	Time
DDoS_HTTP	10
DDoS_UDP	09
DDoS_TCP	08
DoS_TCP	04
DoS_UDP	05
DoS_HTTP	04
Keylogging	04
Data_Exfiltration	02
Reconnaissance	05
Normal	01

**Table 3:** Processed data statistics.

Category	Attack Class	Normal Class	Attributes
Service_Scan	958658	1254954	90
Data Ex-filtration	321542	124512	69
DDoS-HTTP	542154	452145	69
OS-Scan	124585	124562	69
DDoS-TCP	542154	325623	71
Key-logging	145214	215421	69
DoS-HTTP	163254	312451	75
DoS-TCP	124854	325484	69
DDoS-UDP	132541	215421	71
DoS-UDP	965845	965845	70

**Table 4:** Hyper-attribute tuning values.

Attributes	Range	Range	Range
No. of neurons	256	512	1024
Learning rate	0.01	0.001	0.0001
No. of hidden layers	3	4	5
Dropout rate	0.2	–	–
No. of epochs	30	60	110
Window size (seconds)	3	4	5
Batch size	64	64	512

**Table 5:** Comparative analysis of percentage of memory reduction.

Class	GMDH (lr)	GMDH (lr-cov)	χ ^2^	MI
Service	90.23	90.25	90.48	90.14
Data Exfiltration	90.23	90.14	90.59	91.25
DDoS-HTTP	91.25	93.21	90.15	91.02
DDoS-UDP	90.15	90.23	90.14	90.14
OS	90.65	90.48	90.65	90.14
DoS-HTTP	90.47	90.65	90.11	90.32
DDoS-TCP	90.48	90.65	90.12	90.87
Keylogging	90.65	90.41	90.54	90.26
DoS-UDP	90.15	90.16	90.16	90.17
DoS-TCP	90.48	90.489	90.65	90.33

**Table 6:** Performance comparative analysis of ML techniques: Bot IoT data.

Model	Data Size	F1 (%)	Accuracy (%)	Precision (%)	Recall (%)	AUC (%)	Delay
RNN	4.1 M	99.5	99.65	99.65	99.01	99.5	401 s
RF	4.1 M	99.2	98.5	98.6	98.45	98.15	241 s
NB	4.1 M	91.23	99.1	98.56	97.56	98.1	10.25 s
Bi-LSTM	4.1 M	99.15	97.56	94.56	96.58	97.45	841 s
SVM	111 K	95.26	96.23	94.25	94.36	94.15	1625 s

**Table 7:** Performance comparative analysis of ML techniques: NSL-KDD data.

Model	Data Size	F1 (%)	Accuracy (%)	Precision (%)	Recall (%)	AUC (%)	Delay
RNN	5.2 M	98.15	97.54	98.54	98.00	98.40	395 s
RF	5.2 M	98.10	97.4	97.5	97.34	97.04	230 s
NB	5.2 M	90.12	98.0	97.45	96.45	97.0	9.14 s
Bi-LSTM	5.2 M	98.04	96.45	93.45	95.47	96.34	830 s
SVM	132 K	94.15	95.12	93.14	93.15	93.04	1152 s

**Table 8:** Performance comparative analysis (N, not applicable).

Ref.	Technique	Accuracy	Precision	F1-Measure	Recall	AUC
[3]	LSTM	98.56	N	N	N	N
[7]	CNN	94.65	N	N	N	N
[11]	RF	94.69	N	96.15	N	N
[12]	RF	95.65	95.12	N	N	N
[13]	DT	94.56	96.25	94.15	94.56	N
[14]	BiLSTM	94.01	93.2	91.12	N	N
This paper	RNN + BiLSTM	99.56	99.45	99.12	98.25	96.25

**Table 9:** Comparative analysis of number of floating point operations.

	Full	GMDH-LR	GDMH-LRCOV	Chi	MI
Service	1.25	1.02	1.02	1.03	1.07
DDoS-UDP	1.2	1.06	1.07	1.056	1.065
Data-exfiltration	1.23	1.026	1.02	1.06	1.05
OS	1.32	1.02	1.05	1.02	1.02
DoS-UDP	1.14	1.02	1.02	1.02	1.02
Keylogging	1.15	1.04	1.07	1.07	1.02
DoS-TCP	1.2	1.05	1.07	1.02	1.03
DoS-HTTP	1.32	1.07	1.02	1.02	1.04
DDoS-HTTP	1.23	1.065	1.07	1.02	1.02
DDoS-TCP	1.65	1.05	1.04	1.03	1.02

## Data Availability

The data used to support the findings of this study are available from the corresponding author upon request.
